# Effect of Repeated Contact to Food Simulants on the Chemical and Functional Properties of Nano ZnO Composited LDPE Films for Reusable Food Packaging

**DOI:** 10.3390/polym15010009

**Published:** 2022-12-20

**Authors:** Wooseok Lee, Nattinee Bumbudsanpharoke, Gyeong-Hyeon Gawk, Jae-Min Oh, Seonghyuk Ko

**Affiliations:** 1 Department of Packaging and Logistics, Yonsei University, Wonju 26493, Republic of Korea; 2 Department of Packaging and Materials Technology, Faculty of Agro-Industry, Kasetsart University, Bangkok 10900, Thailand; 3 Radwaste Technology Department, Korea Radioactive Waste Agency, Daejeon 38062, Republic of Korea; 4 Department of Energy and Materials Engineering, Dongguk University-Seoul, Seoul 04620, Republic of Korea

**Keywords:** LDPE-ZnO nanocomposite, reusable food packaging, food contact, light barrier, safety

## Abstract

The effect of repeated contact with food simulants on the properties and functionality of zinc oxide (ZnO) in nanocomposite films was investigated to examine possible safety hazards from the point of view of long-term use as food packaging. Low-density polyethylene (LDPE) embedded with 5 wt% nano-ZnO was immersed in distilled water, 50% ethanol, 4% acetic acid, and n-heptane. The cycle of immersion–rinse–dry was repeated up to 40 times for same sample under constant condition. Fourier transform infrared spectroscopy (FTIR), X-ray diffraction (XRD), X-ray absorption spectroscopy (XAS), field emission-scanning electron microscopy (FE-SEM), and UV–Vis spectroscopy analyses were performed to identify the changes in the chemical and functional properties of the nanocomposite film. Acetic acid had the greatest impact on the LDPE-ZnO nanocomposite films, while other food simulants caused little change. A new carboxylate bond was formed by the reaction of ZnO with acetic acid, as evidenced by the FTIR spectra. In addition, XRD and XAS confirmed the phase changes of nano-ZnO into zinc salts such as zinc hydroxy acetate or zinc acetate dihydrate. Furthermore, the light barrier property of the nanocomposite film drastically decreased, owing to the change in the bandgap of ZnO and film morphology.

## 1. Introduction

Recently, nanosized inorganic nanomaterials such as nano-clay, titanium oxide, zinc oxide (ZnO), and silver have been applied to food packaging to endow food packaging with unique properties, such as improved gas and light barrier properties, increased mechanical strength, and enhanced antimicrobial functionality [1,2,3].

ZnO has a wide bandgap of approximately 3.3 eV in the near-UV spectrum, which makes it an excellent UV light absorber in ultraviolet A (UVA, spectral range 315–400 nm) and ultraviolet B (UVB, 280–315 nm) [4,5]. In addition, ZnO has been a well-known antimicrobial agent since the early 1950s [3]. Nanosized ZnO significantly improves the UV barrier and antimicrobial properties of composite films by enhancing the diffusivity and reactivity, owing to its large specific surface area [6]. Silvestre et al. [7] reported extruded isotactic polypropylene (iPP)/ZnO compound films with high antibacterial activity against *Escherichia coli*. Factori et al. [8] assessed the UV-shielding and antimicrobial activity of a ZnO-polyvinyl alcohol composite prepared using the microwave-assisted sol-gel method. They showed that the inhibition zone for Gram-negative and Gram-positive bacteria ranged from 9 to 14 mm, and light absorption was observed at UV wavelengths below 260 nm.

The migration of inorganic nanomaterials from polymer nanocomposites into foodstuffs has been investigated to address human safety concerns. Many studies have discovered that nano-inorganic materials or ions, such as silver [9], titanium dioxide [10], and copper [11], can be leached from packaging to foodstuffs [12]. Similar to other inorganic additives, Zn also migrates into foodstuffs even when at the nanoscale. Panea et al. manufactured an LDPE-Ag, ZnO nanocomposite film, applied it to chicken breast packaging, and detected about 2.44 ppm of migrated zinc in aqueous condition [13]. Lu et al. [14] produced a nano-ZnO-polylactic acid composite film with 1–15% ZnO concentrations. They showed the migration of Zn ions at approximately 50 ppm in 10% ethanol, which was 15 ppm higher than that in iso-octane conditions because of ZnO solubility [14].

Furthermore, it has been reported that the reuse or long-term use of food packaging can promote the migration rate of embedded nanoparticles in the polymer [15]. Heydari-Majd et al. [16] stated that increased levels of Zn migration from nano-ZnO-PLA films to meat fillets were observed during 16 d of storage at 4 °C. Braun et al. [17] reported that the silver doping on polyethylene terephthalate bottles migrated proportionally over a test time of 15 d into a water simulant of up to 50 μg/L, and a silver concentration of 30 μg/L was still observed with the reused condition one year later. Therefore, the increased food contact time because of reuse accelerates the migration effects.

Moreover, several studies have shown that migration may lead to changes in the properties of nanocomposite films. Xia et al. [18] studied the effects of a food simulant on nano-clay composite films. They found that the collapse of the clay gallery diminished the d-spacing of clay, owing to the migration of the surfactant and polymer chain relaxation. Fan, et al. [19] suggested that the migration of Ag from a PLA-Ag composite film induced a decrease in the water barrier and mechanical properties. Our previous study showed that the detachment or dissolution of nano-ZnO from the host matrix results in the loss of the UV-light barrier property [20]. 

This study aimed to investigate the effect of food simulants and time of use on the properties and functionality of LDPE-ZnO nanocomposites according to Korean standards and specifications for food utensils, containers, and packages [21]. The cycle of immersion–rinse–dry was conducted repeatedly under consecutive cycles (1, 5, 20, and 40 cycles) on a single sample to mimic the repeated use of food packaging or containers. The chemical bonds, phase transformation, surface morphology, and UV-light barrier function of the tested LDPE-ZnO nanocomposite film were comprehensively evaluated to determine the potential safety risks in long-term repeated use.

## 2. Materials and Methods

### 2.1. Materials

Nano-ZnO with a diameter of 20–50 nm was purchased from Nano and Future Life (Seoul, Republic of Korea). Zinc acetate hydrate was obtained from Junsei Chemical Co. (Chuo-ku, Tokyo, Japan). The LDPE resin (Lutene LB5000, LG Chemical Co., Yeosu, Republic of Korea) with a melt flow index of 5.0 g/10 min was used for the masterbatch. The other LDPE resin (Lutene LB5000, LG Chemical Co., Yeosu, Republic of Korea) with a melt flow index of 7.5 g/10 min was selected for extrusion film. Distilled (DI) water was obtained from the Human Power I+ water purification system (Human Co., Seoul, Republic of Korea). Ethanol (EtOH, 94–96%), acetic acid (AC, >99.7%), and normal heptane (Heptane, 99%) were acquired from Alfa Aesar (Ward Hill, MA, USA).

### 2.2. Preparation of LDPE-ZnO Nanocomposite Film

The LDPE resin was compounded with nano-ZnO powder into a 10 wt% compositions of masterbatch using a twin-screw extruder (BA-19, BauTek Co., Uijeongbu, Republic of Korea) with a length/diameter (L/D) ratio of 40:19. Subsequently, nanocomposite films were prepared with 5 wt% ZnO content by a melt-extrusion method using a single-screw extruder (Hankook EM Ltd., Pyeongtaek-si, Republic of Korea) with an L/D ratio of 30:30. Before the extrusion, LDPE and nano-ZnO masterbatch pellets were dried in a convection oven at 80 °C for 24 h and blended at the specific mixing ratios of 1:0 and 1:1, respectively. The final concentration of the nanocomposite film was 5 wt% ZnO content. The processing temperature was adjusted to 200–230 °C at a screw speed of 40 rpm. Film thickness was maintained at 80 ± 5 μm.

### 2.3. Repeated Use Test

The experimental method was designed to mimic the repeated use of food packaging or containers by modifying the concept of European Commission No 10/2011: ANNEX V 2.1.6 [22]. This concept was originally used to carry out a migration test on the same specimen to imitate repeated use. For contact conditions when using food simulants, it is assumed that the packaging contacts the same type of food multiple times. A single use was implemented by conducting sample immersion in the different food simulants given in the current Korean regulations [21]. DI water, 4% AC, 50% EtOH, and n-heptane were used as food simulants representing food with pH > 5, food with pH ≤ 5, alcoholic food, and fatty food, respectively. The pH value of each food simulant except n-heptane was 6.5, 2.4, and 6.0. First, film samples were trimmed by cutting them into dimensions of 5 × 5 cm^2^ and were dipped in a 50 mL glass vial filled with a food simulant, which was pre-heated to 70 °C at a rate of 2 mL/cm^2^ surface area. Subsequently, the closed vial was maintained at 70 °C for 30 min in a dry oven. When the n-heptane was used, the sample was maintained at 25 °C for 1 h. After the immersion, each specimen was washed with DI water and dried at 25 °C. A series of immersions, washing, and drying was presumed to be equivalent to a single use of packaging. The treated samples were then repeatedly reprocessed using the same procedure to simulate repeated use and food contact. The multi-treated samples were referred to as 5, 20, and 40 cycles, depending on the number of processes. Finally, all the treated samples were taken out to characterize and evaluate their properties and functionalities compared with those of the fresh film, which was used as the control.

### 2.4. Characterizations

Fourier transform infrared spectroscopy (FTIR) spectra were recorded using a PerkinElmer Spectrum 65 spectrophotometer (PerkinElmer Co., Ltd., Waltham, MA, USA) in the attenuated total reflection (ATR) mode with a C/ZnSe crystal. Each spectrum was obtained in transmittance mode with eight scans and a resolution of 2 cm^−1^ in the wavenumber range of 400–4000 cm^−1^.

The phase characterization of ZnO was performed by X-ray diffraction (XRD) using a Bruker D2 Phaser X-ray diffractometer (Bruker, Billerica, MA, USA) with Ni-filtered Cu Kα radiation (λ = 1.5406 Å). The angular range was 2θ = 5–40° with an angular increment of 2° and an accumulation time of 1 s.

The surface and cross-sectional morphology of the film were observed using field emission-scanning electron microscopy (FE-SEM, Quanta FEG 250, FEI Technologies Inc., Hillsboro, OR, USA). Before analysis, the specimens were sputtered with platinum/palladium using a Cressington Sputter Coater 108 auto (Cressington Scientific Instruments, Watford, UK). Elemental analysis was performed using an energy-dispersive X-ray spectrometer (EDS, Ametek, Berwyn, PA, USA) integrated with FE-SEM. 

X-ray absorption spectroscopy (XAS) of the Zn K-edge was performed at the 8C Nano XAFS beamline at the Pohang Accelerator Laboratory, Pohang, Republic of Korea. The beam size was 0.5 × 1 mm (vertical × horizontal), and the samples were measured in transmission mode. The spectra were normalized with default parameters and Fourier-transformed using the Athena program of the IFEFFIT 1.2.12 software package.

The optical properties of the films were determined by UV–Vis diffuse reflectance spectroscopy using a double beam-V-600 spectrophotometer (Jasco, Tokyo, Japan) with an attached integrating sphere in the wavelength range 200–800 nm.

## 3. Results and Discussion

**Figure 1:**
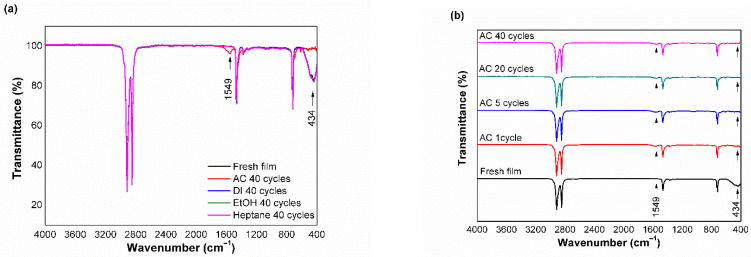
FTIR spectra of (**a**) fresh film and films treated with DI water, EtOH, AC, and heptane after 40 cycles; (**b**) films treated with AC after different number of cycles.

Figure 1a shows the FTIR spectra of the fresh film and films tested with DI water, EtOH, AC, and n-heptane after 40 cycles. The peaks at 2937, 2896, 1458, 1376, and 720 cm^−1^ were identified in all samples as CH_2_ anti-symmetric deformation, CH_3_ symmetric stretching, CH_2_ symmetric deformation, CH_3_ symmetric deformation, and CH_2_ rocking vibration, respectively. These are characteristic peaks of LDPE [23,24]. The peak at 434 cm^−1^ was assigned to the Zn-O stretching vibration, confirming the presence of ZnO in the composite films, and it remained intact even after 40 cycles of exposure to DI water, 50% EtOH, and Heptane. In contrast, this peak completely disappeared from the spectra of the sample immersed in 4% AC, while a new peak at 1549 cm^−1^ was observed, which corresponded to the antisymmetric COO^−^ stretching vibration [25,26]. This finding implies that the embedded nano-ZnO was degraded by AC, especially the carboxylic acid groups from acetic acid, and produced acetate compounds. Yin and Casey [27] reported a similar peak attributed to the carboxylate groups in zinc acetate dihydrate.

In addition, further studies investigated the influence of acidic foodstuff on the stability of nano-ZnO in the composite film by reducing the number of test cycles to 20, 5, and 1 [20]. Figure 1b reveals that the peak at 1549 cm^−1^ appeared after the first repeated use test, whereas the peak at 434 cm^−1^ completely diminished during the first cycle. This result clearly demonstrates that the nano-ZnO particles are highly reactive and chemically unstable with acetic acid.

**Figure 2:**
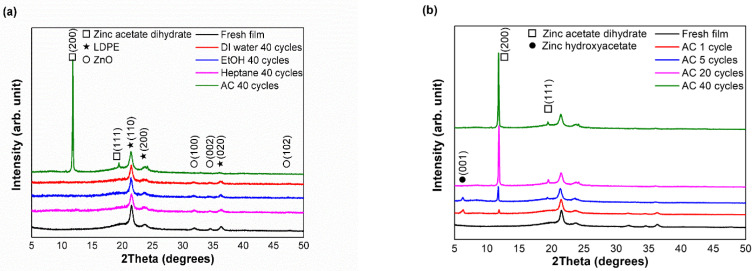
XRD patterns of (**a**) fresh film and films treated with DI water, EtOH, AC, and heptane after 40 cycles; (**b**) XRD patterns of films treated with AC after different number of cycles.

Figure 2a shows the XRD patterns of the fresh film and films obtained after 40 cycles of testing against all food simulants. Diffraction peaks were observed in all samples at 2θ = 21.4°, 23.7°, and 36.5°, representing the orthorhombic crystal structure of LDPE [28]. Furthermore, the peaks at 31.6°, 34.4°, 36.2°, and 47.5° correspond to wurtzite structured zincite (JCPDS No. 36-1451) exhibited by all samples, excluding the sample treated with AC. Moreover, the nano-ZnO composite film treated with AC exhibited new peaks at 11.8° and 19°, which correspond to zinc acetate dihydrate (Zn(CH_3_COO)_2_·H_2_O) (JCPDS No. 33-1464) [29]. This result indicates the phase transformation of ZnO upon contact with the acidic food simulant.

XRD analyses of the films obtained after different number of cycles with AC are shown in Figure 2b. Peaks corresponding to zinc acetate dihydrate were observed in all samples, which were due to the reaction between ZnO and acetic acid. This chemical reaction synthesizes zinc acetate dihydrate, as shown in Equations (1) and (2). The presence of an XRD peak at 6.4° was also identified as isomorphous to hexagonal zinc hydroxy acetate (Zn_5_(OH)_8_(CH_3_COO)_2_) according to JCPDS No. 56-0569 [30]. Some zinc hydroxy acetate can be precipitated from the reaction between zinc acetate and ZnO particles (Equation (3)) [31]. In contrast, the zinc hydroxy acetate peaks disappeared from the samples obtained after 20 and 40 cycles. Zinc hydroxy acetate, which is vulnerable to acidity similar to other zinc salt compounds [32], might have been dissolved in the acidic solution as the number of tests increased. This finding precisely supported the FTIR results, in which carboxylate compounds were generated in the LDPE matrix after first exposure to acetic acid.

(1) $$ZnO+2\;CH_{3}COOH\to \;Zn{\left(CH_{3}COO\right)}_{2}+H_{2}O$$

(2) $$Zn{\left(CH_{3}COO\right)}_{2}+2H_{2}O\to Zn{\left(CH_{3}COO\right)}_{2}\;\cdot \;2H_{2}O$$

(3) $$4ZnO+Zn{\left(CH_{3}COO\right)}_{2}+4H_{2}O\to \;Zn_{5}{\left(OH\right)}_{8}{\left(CH_{3}COO\right)}_{2}$$

**Figure 3:**
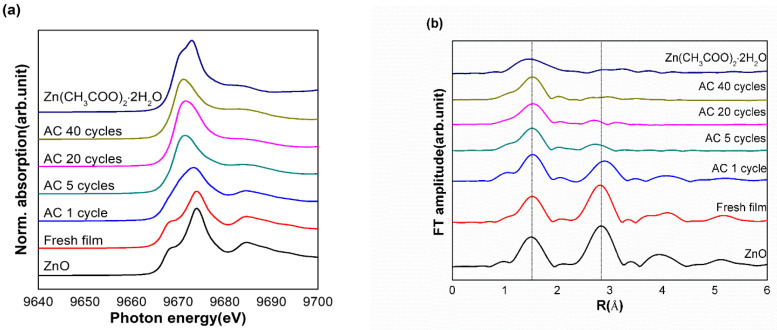
(**a**) XAS spectra of zinc acetate dihydrate and films treated with AC after different number of cycles. (**b**) FT-EXAFS spectra of zinc acetate dihydrate and films treated with AC after different number of cycles.

To verify the local structural changes around the Zn atoms in the LDPE-ZnO nanocomposite films, an XAS supplemental analysis was conducted. Because this spectrum is strongly influenced by chemical properties such as local symmetry, types of ligand, and oxidation state, it is helpful to examine the structural changes of the chemicals. As presented in Figure 3a, a local structural change around Zn in the LDPE-ZnO nanocomposites was detected in the normalized X-ray absorption near edge structure (XANES) of the Zn K-edge. Generally, ZnO with a wurtzite structure showed the main white line (an intense absorption of X-rays near the edge) with a subpeak around 9668 eV due to the 1s → 4p transition [33]. This revealed that ZnO in the LDPE-ZnO nanocomposite film had the same local crystal structure around the Zn atoms as free ZnO. The loss of the subpeak and change in the white line appeared after one cycle of the repeated-use test. The spectrum of the LDPE-ZnO nanocomposite after 40 cycles ultimately showed a similar pattern to that of zinc acetate dihydrate with the main edge at 9664 eV. This result was attributed to the phase transformation from wurtzite ZnO to zinc acetate dihydrate, whcih corresponds to the XRD patterns.

As presented in Figure 3b, the Fourier transformed-extended X-ray absorption fine structure (FT-EXAFS) spectra distinctly exhibited the phase transformation of ZnO in the composite film. The R-space EXAFS spectrum provides information on the bonding distances with neighboring atoms and the electron density of neighboring atoms. The spectrum showed first and second shells at 1.50 and 2.83 Å (non-phase shift corrected), respectively, attributed to Zn-O and Zn-Zn bonds. After one cycle of the repeated use test, the magnitude of the second shell significantly decreased, owing to the local structural change from ZnO to zinc acetate dihydrate. As the number of tests was increased, the second shell in the FT-EXAFS spectrum gradually disappeared. Finally, after 40 cycles, only the first shell was observed from zinc acetate dihydrate [34,35]. Therefore, considering the local structure and coordination environment around Zn in the FT-EXAFS spectra, the transformation of ZnO into another phase was confirmed.

Although ZnO is generally recognized as safe (GRAS), recent studies have shown the possible toxicity of nano-ZnO [36]. Liao et al. [37] reported that nano-ZnO exhibits cytotoxicity and genotoxicity in mammalian cells by generating reactive oxygen species (ROS) and Zn^2+^ ions. Nano-ZnO migrates into the cell to produce ROS, attacking mitochondria, proteins, and DNA molecules, leading to oxidative stress and apoptosis [38,39]. In addition, Zn^2+^ ions dissolved from nano-ZnO disrupt DNA integrity and help generate ROS by suppressing defense compounds against oxidative stress [40]. Furthermore, the National Institute of Health of U.S [41] restricts daily zinc intake to 40 mg/day to prevent acute and chronic toxicity. The toxicological profile of zinc [42] showed that long-term intake of zinc compounds could cause undesirable hematological, hepatic, and renal effects. Moreover, in oral exposure, the LD50 value of zinc acetate was 186 mg/kg/day, which is the lowest value among the zinc compounds.

XRD patterns and XAS spectra showed that a zinc salt was formed in the nanocomposite film matrix (i.e., zinc acetate dihydrate or zinc hydroxy acetate). It is well known that zinc acetate compounds are more soluble in water and alcohol than ZnO [32]. Therefore, this phase transition of ZnO in the nanocomposite film boosts the migration of Zn^2+^ ions into foodstuffs. Our previous study also proved that the leaching level of Zn^2+^ ions from the composite film is increased at a lower pH of contact fluids, that is, 4% acetic acid [20]. The possibility of excessive daily intake of Zn substrate should be considered when applying nano-ZnO to acidic food packaging.

**Figure 4:**
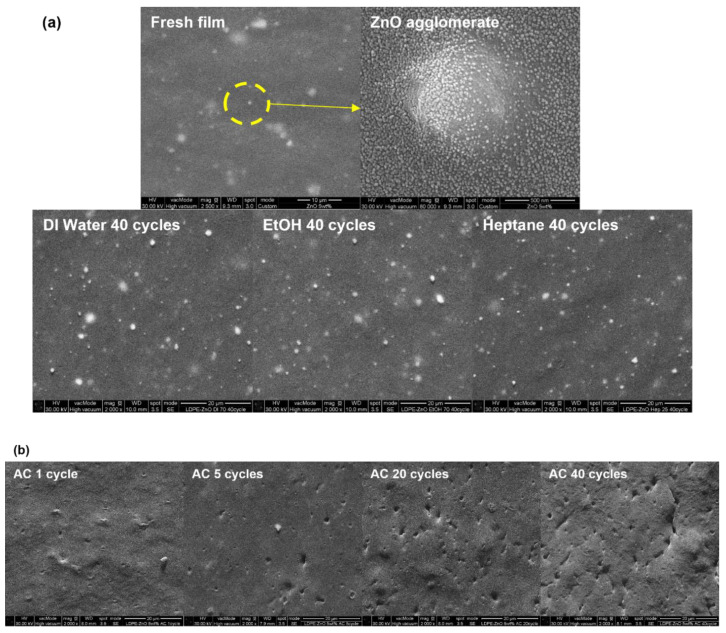
FE-SEM images of (**a**) fresh film and films treated with DI water, EtOH, and heptane. (**b**) Surface of films treated with AC after different number of test cycles.

Figure 4a illustrates the surface morphology of the LDPE-ZnO composite films treated for 40 cycles with the food simulants. The micrograph of the fresh film shows white dots of dispersed nano-ZnO particles as aggregates on the surface, and the diameter of their clusters was approximately 1–3 μm. Even though the films repeatedly contacted DI water, EtOH, and heptane, nano-ZnO particles were still present on the surface of the films. In contrast, the nano-ZnO particles vanished after only the first cycle of exposure to 4% AC, as shown in Figure 4b. The detachment of the nano-ZnO particles results deep and extensive pores, which increases with the number of test cycles. As shown by FTIR, XRD, and XAS, ZnO transforms to zinc acetate or zinc hydroxy acetate, and this compound might be leached out from the surface during the test. Polat et al. [43] reported a similar result by showing the surface erosion and holes on the ZnO composite film after contact with a 3% AC solution at 40 °C for 10 d.

**Figure 5:**
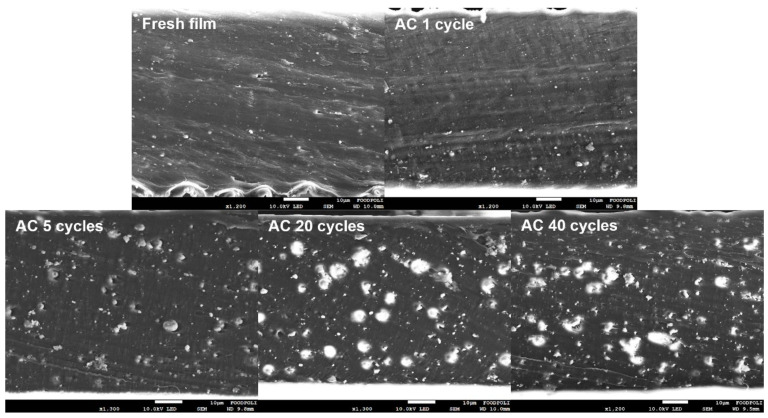
FE-SEM images of the cross-section of films treated with AC after different number of test cycles.

Figure 5 shows the cross-section of the tested composite films taken from different cycles under AC conditions. Dispersed ZnO agglomerates were also observed on the surface of the nanocomposite films, in addition to the interior of the fresh nanocomposite films. However, the size of the zinc compounds significantly increased as the number of repetitions increased, which was attributed to the transformation of ZnO into zinc acetate dihydrate. The chemical structure of wurtzite ZnO is a mineral crystal closely bound to one Zn atom and four O atoms; the O atom bonds with the other four Zn atoms. In the zinc acetate dihydrate structure, one Zn atom is bonded to two bidentate acetate ligands and two H_2_O molecules by a coordination bond [44]. Consequently, the phase transformation of ZnO augmented the number of bonded atoms per Zn atom from 4 to 20, which increased the mass and size of the zinc compounds.

**Figure 6:**
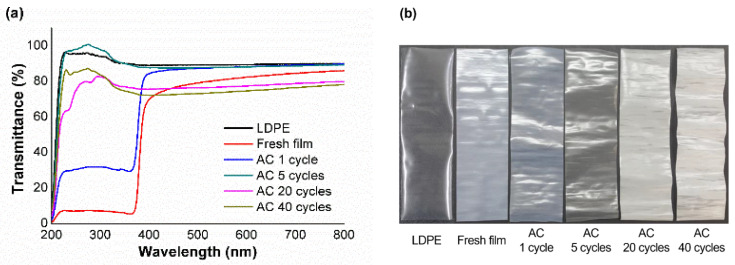
UV–Vis spectra (**a**) and digital photographs (**b**) of fresh film and LDPE-ZnO nanocomposite films treated with AC after different number of test cycles.

The results in Figure 6a show that nano-ZnO particles strongly enhanced the UV-light barrier of the LDPE film. The nanocomposite film allowed 7% of light to pass in the UV range (200–370 nm), which is approximately 88% lower than that of a pure LDPE film. However, the UV-light barrier property was diminished drastically by AC after the first exposure, and the transparency in the UV-light range increased with the number of test cycles. Figure 6b represents the appearance of the films that were gradually whitened as the number of repetitions increased with acetic acid. This was mainly attributed to the transformation of ZnO, as shown in Figure 2 and Figure 3. The hybridized ionic and covalent bonds were formed by coordination between Zn and O ions in the wurtzite structure, which makes the semiconductor characteristic of ZnO similar to a bandgap [45]. Therefore, zinc acetate or zinc hydroxy acetate loses UV absorption property because the Zn-O band has been replaced by other chemicals, such as acetate and a hydroxyl group [44].

## 4. Conclusions

In this study, a repeated use test was conducted to evaluate the effects of the multiple usages on the chemical and functional properties of LDPE-ZnO nanocomposite film for reusable food packaging. To simulate the repeated use of packaging, sample were immersed in food simulants, rinsed, and dried repeatedly for up to 40 cycles. As a result, only 4% acetic acid, representing acidic foods, caused the most significant chemical change in ZnO, while the other simulants were harmless. With repeated contact with 4% acetic acid, the chemical bonds of Zn-O disappeared, and the wurtzite structure of ZnO was completely transformed to zinc acetate dihydrate and zinc hydroxy acetate. Moreover, the destruction of the bandgap of ZnO by chemical structure variation caused the loss of the UV barrier property of the nanocomposite film. Furthermore, zinc compounds transformed by reaction with acetic acid have a higher solubility than ZnO, which could increase the possibility of daily zinc intake through acidic food. It is noteworthy that functional and safety considerations are critical when applying ZnO to food packaging, particularly for acidic foods.

## Data Availability

The original contributions presented in the study are included in the article, further inquiries can be directed to the corresponding author.
